# CCND1 Splice Variant as A Novel Diagnostic and Predictive Biomarker for Thyroid Cancer

**DOI:** 10.3390/cancers10110437

**Published:** 2018-11-13

**Authors:** Sora Jeon, Yourha Kim, Young Mun Jeong, Ja Seong Bae, Chan Kwon Jung

**Affiliations:** 1 Department of Hospital Pathology, College of Medicine, The Catholic University of Korea, Seoul 06591, Korea; thfk38@nate.com (S.J.); chuchura@naver.com (Y.K.); dydrkf1993@naver.com (Y.M.J.); 2 Department of Biomedicine & Health Sciences, Graduate School, The Catholic University of Korea, Seoul 06591, Korea; 3 Cancer Research Institute, College of Medicine, The Catholic University of Korea, Seoul 06591, Korea; drbae@catholic.ac.kr; 4 Department of Surgery, College of Medicine, The Catholic University of Korea, Seoul 06591, Korea

**Keywords:** *CCND1*, cyclin D1, *CCND1b*, cyclin D1b, thyroid cancer, papillary thyroid carcinoma, noninvasive follicular thyroid neoplasm with papillary-like nuclear features, NIFTP, BRAF, polymorphism

## Abstract

Cyclin D1 protein is aberrantly overexpressed in thyroid cancers, but mutations of the *CCND1* gene are rare in these tumors. We investigated the *CCND1* rs9344 (G870A) polymorphism and the expression profiles of wild-type *CCND1a* and shortened oncogenic isoform *CCND1b* at the mRNA and protein levels in 286 thyroid tumors. Genotype AA of rs9344 was associated with high expression of *CCND1b* mRNA and was more frequently found in thyroid cancer than in benign tumors. The mRNA expression levels of *CCND1b* were higher in papillary thyroid carcinoma (PTC) than in benign or other malignant tumors. However, the expression of *CCND1a* mRNA showed no association with the parameters. Noninvasive follicular thyroid neoplasm with papillary-like nuclear features (NIFTP) was distinguished from PTC by low expression of *CCND1b* at mRNA and protein levels. We further observed that cyclin D1b immunostaining helped to avoid the misdiagnosis of classic PTC with predominant follicular pattern as NIFTP in a separate cohort. Nuclear cyclin D1b expression was associated with aggressive clinicopathologic features in PTC. These findings suggest that cyclin D1b overexpression can be used as a diagnostic and predictive biomarker in thyroid tumors and may be functionally involved in the development and progression of the disease.

## 1. Introduction

Thyroid cancers constitute the majority of endocrine malignancies. Differentiated thyroid cancers, arising from follicular cells, constitute more than 95% of all thyroid cancers and are histologically classified as either papillary thyroid carcinoma (PTC), follicular thyroid carcinoma, or poorly differentiated thyroid carcinoma [1]. The 2017 World Health Organization Classification of Tumors of Endocrine Organs incorporates the newly defined entity noninvasive follicular thyroid neoplasm with papillary-like nuclear features (NIFTP), which is a neoplasm with an unspecified, borderline, or uncertain clinical behavior, but not a benign or malignant tumor [1,2]. Anaplastic thyroid carcinoma also arises from follicular cells but is a highly aggressive disease, while differentiated thyroid cancer is generally considered an indolent disease. Medullary thyroid carcinoma, a tumor derived from parafollicular C cells (neural crest origin), has biologic features that differ from those of follicular cell-derived cancers. The incidence of thyroid cancer has been increasing steadily worldwide over the last few decades [3,4,5,6]. The most rapid rise in the incidence of thyroid cancer was observed in South Korea, where the rate increased nearly 15-fold from 1993 to 2011 [5,7]. This increase is primarily attributed to the rising incidence of PTC, and specifically to that of low-risk PTC including subcentimeter-sized cancer or encapsulated follicular variant [4,7,8]. PTC accounts for 85% of thyroid cancers in most countries, whereas in Korea PTC accounts for more than 95% of thyroid cancers (www.cancer.go.kr) [7,8]. Differentiated thyroid cancers are generally considered indolent; therefore, there is a need to develop preoperative diagnostic markers that can be used to identify thyroid cancers requiring surgical removal from thyroid nodules.

The cyclin D1 protein, coded by the *CCND1* gene, is a gate-keeper regulating the transition from the G1 phase into the S phase of the cell cycle [9,10]. Overexpression of cyclin D1 is observed in a variety of human cancers and is involved in tumorigenesis [9,10]. The overexpression of cyclin D1 in human cancers can result from genetic alterations, changes in epigenetic regulation, gene transcription, and protein translation of *CCND1*. We have shown that cyclin D1 is consistently overexpressed in PTC, and cyclin D1 immunostaining is useful for identifying the extent of tumor involvement [11]. However, mutations and amplification of the *CCND1* gene have rarely been found in the differentiated thyroid cancer [12,13]. The *CCND1* gene encodes two major splice variants: wild-type (*CCND1a* mRNA) and an oncogenic isoform (*CCND1b* mRNA) [9]. *CCND1a* consists of five exons containing a coding DNA sequence of 888 bp, which encodes a 295-amino acid protein (cyclin D1a). Failure to splice at the exon 4-intron 4 boundary of the *CCND1* pre-mRNA generates the *CCND1b* splice variant that contains intron 4 [9]. Because intron 4 contains a translation stop codon, *CCND1b* lacks exon 5 and encodes a 275-amino acid protein (cyclin D1b) with early termination of transcription at the intron 4 region [9]. Degradation of cyclin D1 is regulated by C-terminal PEST domain and threonine residue 286 [10]. However, the absence of a protein-destabilizing (PEST) domain and threonine residue 286 in cyclin D1b suggests that cyclin D1b is regulated by a different mechanism and is more stable than cyclin D1a [10]. Polymorphism rs9344 (G870A) at the critical exon 4 splice junction is associated with the expression of *CCND1b* [9]. The G allele at nucleotide 870 (codon 242) preferentially encodes the *CCND1a* transcript, and the A870 allele preferentially encodes *CCND1b* [14]. However, expression of *CCND1b* can be found in tumors homozygous for G/G [15]. Cyclin D1b expression is related to tumorigenesis, tumor progression, and poor outcomes in various human cancers such as those of the brain, esophagus, lung, breast, colon, prostate, and bladder, as well as in Ewing sarcoma and lymphoma [9,14,15,16,17,18,19,20,21,22,23,24,25]. However, the roles of cyclin D1b expression and G870A polymorphism in thyroid cancer have not been fully defined.

In this study, we evaluated the diagnostic and clinical utility of mRNA and protein expression of *CCND1* isoforms in thyroid tumors, and assessed the correlation between G870A polymorphism and expression of *CCND1*. We also investigated using the expression of cyclin D1b to differentiate NIFTP from benign thyroid tumors and PTC.

## 2. Results

### 2.1. CCND1 G870A Polymorphism (rs9344) and CCND1 mRNA Expression

**Figure 1:**
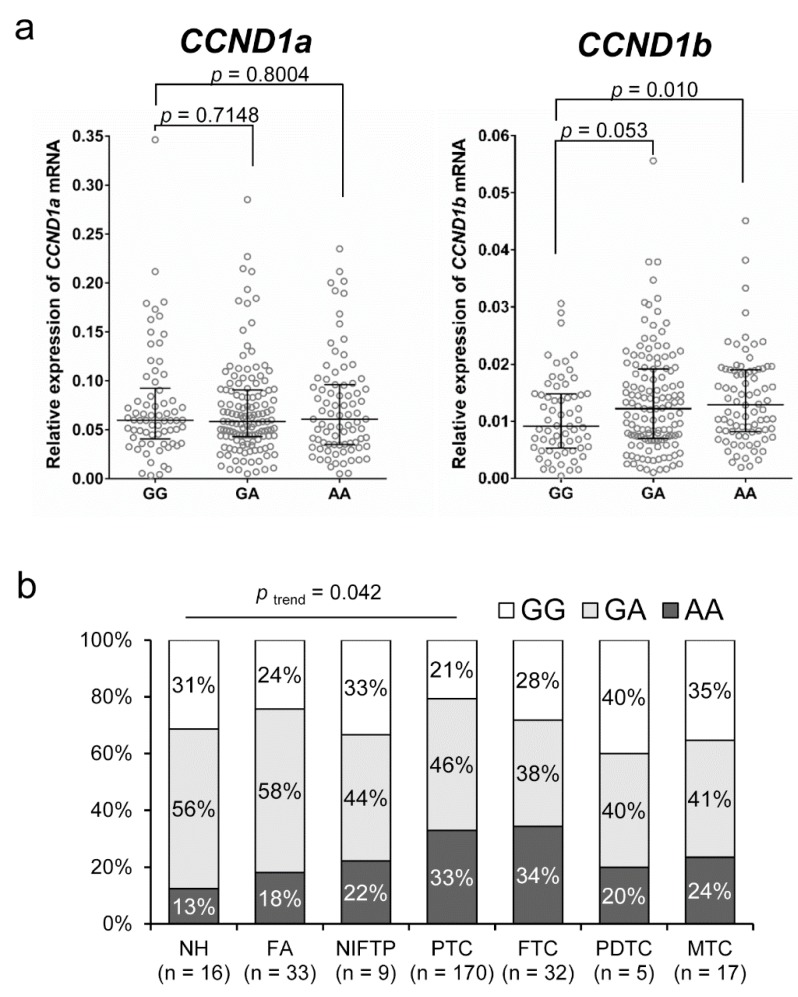
Association of *CCND1* G870A polymorphism (rs9344) and the levels of *CCND1* mRNA expression in thyroid tumors. (**a**) The AA genotype is associated with high levels of *CCND1b* mRNA but not with those of *CCND1a* mRNA. (**b**) The distribution of rs9344 genotypes with respect to the various types of thyroid tumors. NH, nodular hyperplasia; FA, follicular adenoma; NIFTP, noninvasive follicular thyroid neoplasm with papillary-like nuclear features; PTC, papillary thyroid carcinoma; FTC, follicular thyroid carcinoma; PDTC, poorly differentiated thyroid carcinoma; MTC, medullary thyroid carcinoma.

The *CCND1* G870A genotypes were significantly correlated with mRNA expression of *CCND1b* but not with expression of *CCND1a*, as shown in Figure 1a. The level of *CCND1b* expression was significantly higher in the genotype AA group than in the genotype GG group (*p* = 0.010). As shown in Figure 1b, there was a trend toward increasing percentage of the AA genotype with increasing disease aggressiveness from nodular hyperplasia to well differentiated thyroid cancer (*p* trend = 0.042).

### 2.2. Relationship between the mRNA Expression of CCND1 Isoforms and Types of Thyroid Tumor

**Figure 2:**
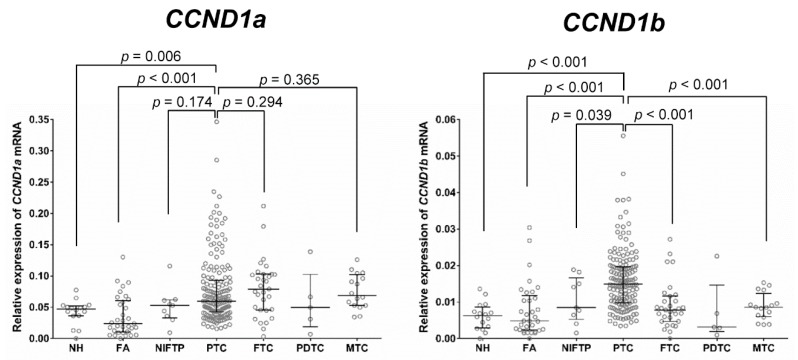
Expression of *CCND1* mRNA isoforms relative to that of *GAPDH* in various types of thyroid tumors. NH, nodular hyperplasia; FA, follicular adenoma; NIFTP, noninvasive follicular thyroid neoplasm with papillary-like nuclear features; PTC, papillary thyroid carcinoma; FTC, follicular thyroid carcinoma; PDTC, poorly differentiated thyroid carcinoma; MTC, medullary thyroid carcinoma.

Figure 2 shows the mRNA expression level of *CCND1* isoforms according to the type of thyroid tumor. The expression of *CCND1a* and *CCND1b* mRNA was significantly increased in PTC compared to that in benign lesions (nodular hyperplasia and follicular adenoma). NIFTP showed a lower level of *CCND1b* mRNA expression than that observed in PTC (*p* = 0.039). However, there was no difference in the expression level of *CCND1a* mRNA between NIFTP and PTC (*p* = 0.174). No significant differences in the expression levels of *CCND1a* and *CCND1b* mRNA were found among nodular hyperplasia, follicular adenoma, and NIFTP. Follicular thyroid carcinoma and medullary thyroid carcinoma showed a lower expression level of *CCND1b* mRNA than that of PTC (*p* < 0.001). However, there were no differences in the expression level of *CCND1a* mRNA among PTC, follicular thyroid carcinoma, and medullary thyroid carcinoma.

### 2.3. Protein Expression of Cyclin D1 Isoforms in Different Types of Thyroid Tumors

**Figure 3:**
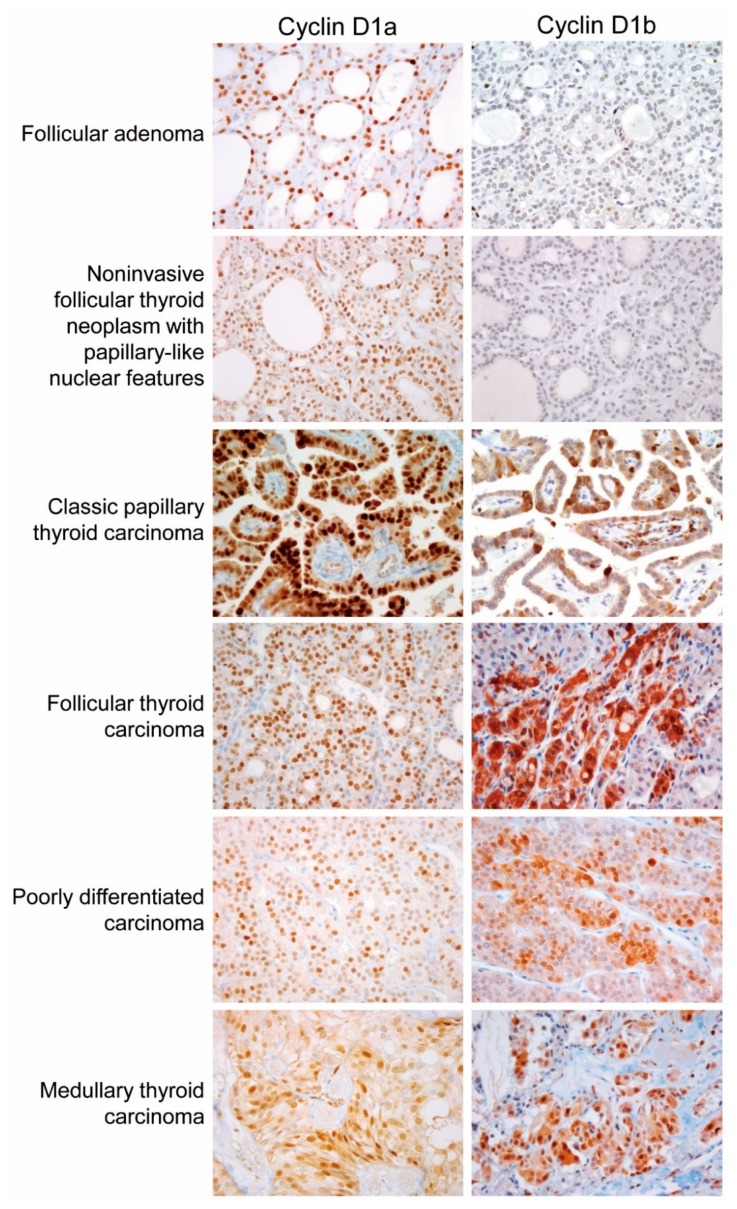
Immunohistochemical staining of cyclin D1a and cyclin D1b in various types of thyroid tumors (×400).

**Figure 4:**
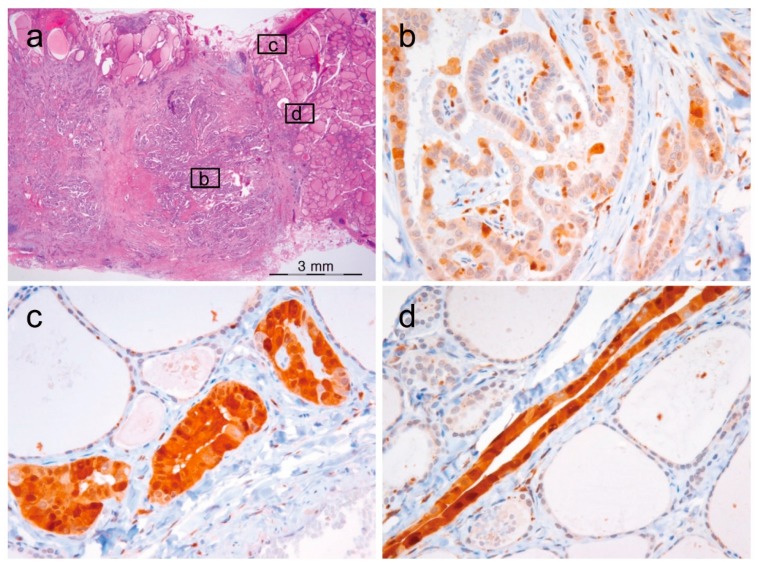
Heterogeneous immunostaining for cyclin D1b in papillary thyroid carcinoma. (**a**) Low magnification (×12) shows a classical papillary thyroid carcinoma with insets corresponding to high-power view of immunostaining for cyclin D1b shown in (**b**–**d**). (**b**) The central areas show a patchy staining pattern (×400). (**c**–**d**) Tumor cells at the invasive front show a diffuse staining pattern (×400).

The expression of cyclin D1a was observed in a great majority of tumor cells (50–100%) in NIFTP, PTC, follicular thyroid carcinoma, poorly differentiated thyroid carcinoma, and medullary thyroid carcinoma, as shown in Figure 3. All cases of nodular hyperplasia were negative for the expression of cyclin D1a and cyclin D1b, as shown in Table 1. Positivity for cyclin D1a was observed in 8 (33%) of 33 follicular adenomas and all cases of NIFTP. All cases of nodular hyperplasia and follicular adenoma were negative for cyclin D1b. One (11%) of 9 NIFTP cases showed nuclear expression of cyclin D1b in cohort 1, as shown in Table 1. In PTC, cyclin D1b showed a patchy immunostaining pattern in the central areas of tumor tissue, and diffuse expression in the periphery and at the invasive front, as shown in Figure 4. In other tumors, immunostaining for cyclin D1b showed a patchy pattern of expression regardless of the areas of tumor tissue, as shown in Figure 3. The expression rate of cyclin D1b was significantly lower in the follicular variant of PTC and follicular thyroid carcinoma than in other types of malignant thyroid tumors, as shown in Table 1. The adjacent non-tumor thyroid tissue was negative for both cyclin D1a and cyclin D1b; however, Hürthle cells in Hashimoto’s thyroiditis were strongly positive for cyclin D1a, but negative for cyclin D1b, as shown in Supplementary Figure S1. Parathyroid cells were positive for cyclin D1a, but negative for cyclin D1b.

### 2.4. Clinical Impact of Expression of CCND1 mRNA Isoforms and Cyclin D1b Protein in PTC

The mRNA expression levels of the two *CCND1* isoforms were grouped as high or low based on the median value, as shown in Table 2. High expression of *CCND1a* mRNA was associated with old age (≥55 years; *p* = 0.042), histologic variant (*p* < 0.001), distant metastasis (*p* = 0.028), and advanced stage as designated by the American Joint Committee on Cancer (AJCC) 8th edition (*p* = 0.022). High expression of *CCND1b* mRNA was associated with lymph node metastasis (*p* = 0.047) and advanced stage, designated by AJCC 7th (*p* = 0.001) and 8th (*p* = 0.011) editions. Expression profiles of nuclear and cytoplasmic cyclin D1b are shown in Table 2. The expression of nuclear cyclin D1b was associated with histologic variant (*p* = 0.009), lymph node metastasis (*p* = 0.002), risk of recurrence (*p* = 0.043), and advanced stage as designated by AJCC 7th edition (*p* = 0.047). The expression of cytoplasmic cyclin D1b was associated with histologic variant (*p* < 0.001), lymph node metastasis (*p* < 0.001), and high risk for cancer recurrence (*p* = 0.010).

We further investigated the clinical impact of *CCND1* mRNA and cyclin D1b protein expression in 125 patients with classic type of PTC, as shown in Table 3. High expression of *CCND1a* mRNA was associated with old age (≥55 years; *p* = 0.022), distant metastasis (*p* = 0.007), and advanced stage as designated by AJCC 7th (*p* = 0.014) and 8th (*p* = 0.004) editions. High expression of *CCND1b* mRNA was associated with lymph node metastasis (*p* = 0.038), and advanced stage as designated by AJCC 7th (*p* = 0.007) and 8th (*p* = 0.008) editions. The expression of nuclear cyclin D1b was associated with lymph node metastasis (*p* = 0.006) and advanced stage as designated by AJCC 8th edition (*p* = 0.038). The expression of cytoplasmic cyclin D1b was associated with lymph node metastasis (*p* = 0.005) and advanced stage as designated by AJCC 7th edition (*p* = 0.025).

### 2.5. Expression of CCND1b mRNA and Cyclin D1b Protein in NIFTP and Invasive Encapsulated Follicular Variant of PTC

**Figure 5:**
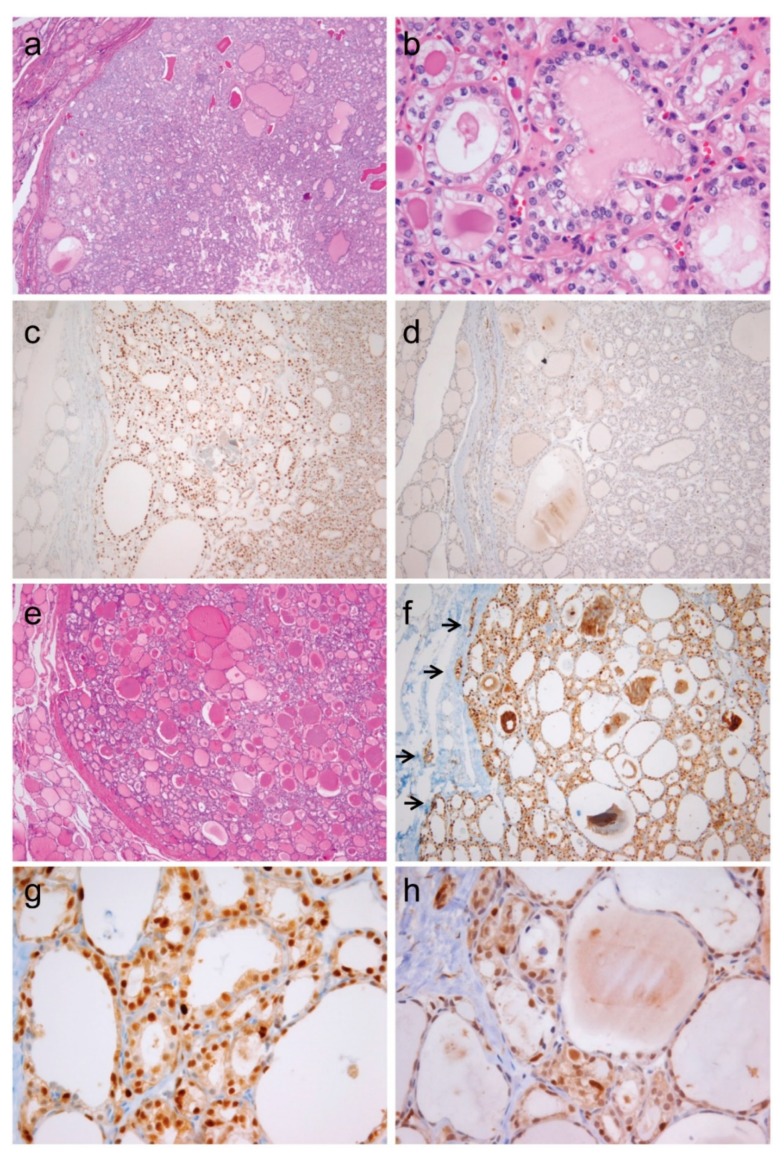
Immunohistochemistry for cyclin D1a and cyclin D1b in noninvasive follicular thyroid neoplasm with papillary-like nuclear features (**a**–**d**) and invasive encapsulated follicular variant of papillary thyroid carcinoma (**e**–**h**). (**a**) Image of noninvasive follicular thyroid neoplasm with papillary-like nuclear features shows clear demarcation and follicular growth pattern (×40). (**b**) This tumor shows the nuclear features of papillary thyroid carcinoma characterized by nuclear enlargement, overlapping, and membrane irregularities (×400). (**c**) Immunohistochemical staining positive for cyclin D1a (**c**, ×100) and negative for cyclin D1b (**d**, ×100). (**e**) Histologic features of an invasive encapsulated follicular variant of papillary thyroid carcinoma (×40). (**f**) Positive immunostaining for cyclin D1a demarcates the site of invasive growth (indicated by arrows, ×100). High magnification (×400) showing positive immunostaining for cyclin D1a (**g**) and cyclin D1b (**h**).

We further evaluated the diagnostic utility of *CCND1b* mRNA and cyclin D1 protein expression in an independent cohort of NIFTP and invasive encapsulated follicular variant of PTC, as shown in Table 4 and Figure 5. There was no difference in the expression of *CCND1b* mRNA between NIFTP and invasive encapsulated follicular variant of PTC, as shown in Table 4. Nuclear expression of cyclin D1b was significantly higher in invasive encapsulated follicular variant of PTC than in NIFTP, as shown in Table 4 (*p* = 0.046). However, there was no difference in the cytoplasmic expression of cyclin D1b between these two diseases (*p* = 0.096).

### 2.6. A Case of Noninvasive Encapsulated PTC with Predominant Follicular Growth and BRAF V600E Mutation

**Figure 6:**
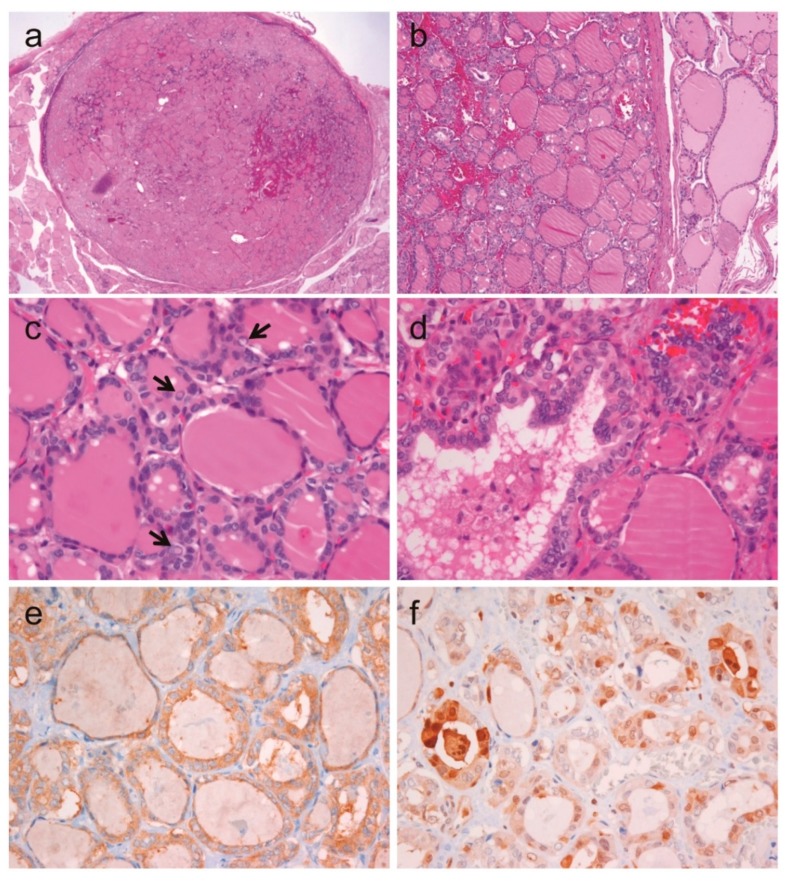
Noninvasive encapsulated classic papillary thyroid carcinoma with predominant follicular growth and *BRAF* V600E mutation. (**a**) Low magnification shows a well-circumscribed follicular tumor measuring 1.1 cm in greatest dimension (×12). (**b**) The tumor is clearly demarcated from normal thyroid tissue by a fibrous capsule (×100). (**c**) The nuclear features of tumor cells show nuclear enlargement, membrane irregularities, pseudoinclusions (indicated by arrows), and grooves (×400). (**d**) Follicles with abortive papillae are observed (×400). Immunohistochemistry shows diffuse positivity for BRAF VE1 (**e**) and focal positivity for cyclin D1b (**f**).

Among the 175 PTCs in cohort 1, one was a noninvasive encapsulated tumor measuring 1.1 cm in diameter, and showing predominantly follicular growth and less than 1% of papillae, as shown in Figure 6. Tumor cells showed the typical nuclear features of PTC. Immunohistochemistry for BRAF V600E (VE1) showed a diffuse strong cytoplasmic staining. Sanger sequencing for *BRAF* exon 15 confirmed the *BRAF* V600E mutation. This tumor was also positive for cyclin D1b immunostaining. Therefore, this tumor was classified as noninvasive encapsulated classic PTC with predominantly follicular growth according to the revised diagnostic criteria for NIFTP [26].

### 2.7. CCND1 Mutation and mRNA Expression in TCGA Dataset

Genetic alteration of *CCND1* was not found in the Cancer Genome Atlas (TCGA) dataset of PTC. The high expression level of *CCND1* mRNA was correlated with the *BRAF*-like cancer (*p* < 0.001). However, there was no association between *CCND1* mRNA expression and clinicopathologic features, as shown in Supplementary Table S1.

## 3. Discussion

To the best of our knowledge, this study is the first to demonstrate the clinicopathologic significance of mRNA and protein expression of *CCND1* isoforms in thyroid tumors. NIFTP was distinguished from PTC by low expression of *CCND1b* mRNA and protein, whereas the expression level of *CCND1a* mRNA and protein in NIFTP did not differ from that observed in PTC. Cyclin D1b expression, as assessed by immunohistochemistry, was significantly lower in NIFTP than in its closest mimic, which is invasive encapsulated follicular variant of PTC. In PTC, nuclear expression of cyclin D1b was associated with aggressive clinicopathologic features including lymph node metastasis, risk of tumor recurrence, and advanced stage.

The association of *CCND1* rs9344 (G870A) polymorphism and risk of thyroid cancer has been reported in a few studies [27,28]. In the Polish population, the AA genotype was more frequently found in patients with PTC than in the healthy population (23.1% vs. 18.5%). The AA genotype may be a risk factor for the development of this type of cancer (odds ratio, 1.452; 95% confidence interval, 1.059–1.989) [27]. A study on the Turkish population showed that the frequency of the AA genotype was significantly higher in patients with PTC than in healthy individuals (37.3% vs. 28.7%) [28]. In our study, the polymorphism rs9344 was associated with the AA genotype. Frequency of the AA genotype was significantly higher in patients with PTC than in those with nodular hyperplasia (33% vs. 13%), although we did not compare these results with a healthy control group. Nevertheless, the *CCND1* rs9344 (G870A) polymorphism may also be a risk factor for developing PTC in the Korean population; this notion is supported by the agreement of our results with the data obtained from studies of the Polish and Turkish populations.

Previous studies showed that high expression of nuclear cyclin D1 is associated with lymph node metastases in PTCs [29,30]. This association, however, is not consistent. In other studies, lymph node metastasis of PTC was not associated with the intensity or distribution of cyclin D1 immunostaining [31]. In our previous studies, as well as in the present study, we observed that cyclin D1a was consistently overexpressed in PTC and there was no correlation between its overexpression and lymph node metastasis [11]. The expression level of *CCND1a* mRNA also had no impact on lymph node metastasis, as assessed using our study cohort and TCGA dataset. With respect to pathologic diagnosis of thyroid tumors, cyclin D1a immunostaining was useful for the differential diagnosis of non-neoplastic hyperplasia and thyroid neoplasms; this is because nodular hyperplasia was completely negative for the expression of cyclin D1a. However, the expression of cyclin D1a did not show clinically- or diagnostically-significant differences between NIFTP and other types of thyroid cancers, provided that all these tumors exhibited overexpression of cyclin D1a. Previous studies exploring the diagnostic role of cyclin D1a in tumors with a follicular pattern showed no difference in cyclin D1a immunostaining among follicular adenoma, follicular thyroid carcinoma, and follicular variant of PTC [31]. Another study showed the expression of cyclin D1a in nodular goiter [31]. These conflicting results described in the literature, as well as in our studies, may reflect variations in population diversity, cut-off values used for the evaluation of cyclin D1 expression, and various conditions used for performing immunohistochemistry.

With respect to the expression of cyclin D1b in thyroid tumor, no preexisting data were found in the literature. In this study, we showed that cyclin D1b was not expressed in follicular adenoma and rarely expressed in NIFTP. In PTC, 64.7% of the samples were positive for nuclear expression of cyclin D1b, which was associated with tumor metastasis, advanced stage, and increased risk of recurrence. These results are consistent with previous findings showing that as an oncogenic isoform, cyclin D1b plays a role in tumorigenesis and progression, and is correlated with poor outcomes in various non-thyroidal cancers [9,14,15,16,17,18,19,20,21,22,23,24,25]. A recent study showed that knockdown of *CCND1b* promoted apoptosis and suppressed cancer-cell stemness and epithelial mesenchymal transition in human bladder cancer cells [32]. These results indicate that the cyclin D1b oncoprotein may play a role in thyroid cancer progression.

Cyclin D1 is a nuclear protein regulating cell cycle progression from the G1 to the S phase and has been implicated in tumor invasion and metastasis in human cancers [33]. Phosphorylation of threonine residue 286 within the PEST domain enables cyclin D1 nuclear exportation and subsequent ubiquitin-dependent degradation in the cytoplasm [34]. Altered ubiquitin-proteasome system is responsible for cyclin D1 overexpression in tumor cells [10,34]. Cytoplasmic expression of cyclin D1 can control cancer cell migration, invasion, and metastasis, but not cell proliferation [33,35,36]. In this study, cytoplasmic immunoreactivity of cyclin D1a was observed in most thyroid cancers, but rarely found in follicular adenoma and NIFTP. The cytoplasmic expression of cyclin D1b was observed only in thyroid cancers and was associated with lymph node metastasis and advanced stage in PTC patients. Further studies are necessary to elucidate the cytoplasmic cyclin D1-dependent mechanisms that control cell adhesion and migration in thyroid cancer.

In some cases, the initial histologic criteria for NIFTP have resulted in the misdiagnosing of encapsulated classic PTC with predominant follicular growth as NIFTP in some cases [26,37,38,39]. Our previous study showed that several encapsulated follicular patterned tumors with nuclear features of PTC developed micro-metastases in regional lymph nodes or harbored the *BRAF* V600E mutation, when criteria of less than 1% papillae was allowed [37]. In another study, a 6.0-cm thyroid tumor, which met the initial criteria for NIFTP, had concurrent *RAS* and *TERT* promoter mutations [40]. As a result, the diagnostic criteria of NIFTP have recently been updated to avoid misdiagnosing these thyroid cancers as NIFTP [2,26]. The revised diagnostic criteria recommend using the criterion of “no (0%) well-formed papillae” and thorough examination of the whole-tumor capsule to exclude the presence of capsular or vascular invasion [26]. Furthermore, the entire tumor tissue should be submitted for histologic examination to exclude the presence of any papillae when the tumor has florid nuclear features (nuclear score of 3) of PTC [26]. Exclusion criteria include the presence of *BRAF* V600E and *BRAF* V600E-like mutations, or that of high-risk mutations (such as those in *TERT* promoter, *TP53*), even if the tumor meets the histologic criteria for NIFTP. Molecular testing, however, is not mandatory for NIFTP diagnosis. In our present study, one case met the former microscopic criteria for NIFTP [2], but was positive for the *BRAF* V600E mutation and showed positive immunostaining for cyclin D1b and BRAF VE1. Our findings further support the recommended detection of *BRAF* V600E by molecular testing or immunohistochemistry in order to differentiate classic PTC from NIFTP. Immunohistochemical staining for nuclear cyclin D1b can be helpful in diagnosing NIFPT, provided that nuclear cyclin D1b is rarely expressed in NIFTP but is highly expressed in PTC. We also observed a significantly higher nuclear expression of cyclin D1b in invasive encapsulated follicular variant of PTC than in NIFTP, as assessed in an independent cohort. These observations suggest that cyclin D1b immunostaining can be used in distinguishing NIFTP from its closest histologic mimic, invasive encapsulated follicular variant of PTC. In this context, the nuclear positivity for cyclin D1b immunostain indicates that the entire tumor tissue should be evaluated for evidence of malignancy such as true papillae, high-grade features, or invasion.

Various benign thyroid nodules can show atypical nuclear features, mimicking those of PTC. Hashimoto’s thyroiditis is the most common cause of false positive results in preoperative aspiration cytology. Immunostaining for cytokeratin-19, galectin-3, HBME1, and loss of expression l of CD56 are frequently used to diagnose PTC. However, the expression of cytokeratin-19, galectin-3, HBME1, and loss of expression of CD56 are also detected in 20%, 20–40%, 20%, and 7–90% of patients with Hashimoto’s thyroiditis, respectively [41,42]. In the present study, cyclin D1b was not expressed in Hashimoto’s thyroiditis, as shown in Supplementary Figure S1. However, immunostaining for cyclin D1a was strongly positive in the Hürthle cells of Hashimoto’s thyroiditis, as shown in Supplementary Figure S1. These findings suggest that immunostaining for cyclin D1b, rather than for cyclin D1a, can be used to differentiate between Hashimoto’s thyroiditis and PTC in limited biopsy samples or cell blocks.

RNA extracted from formalin-fixed paraffin-embedded (FFPE) tissue blocks has often suffered degradation over time. The quality of RNA derived from FFPE samples is affected by pre-analytical procedures including time to fixation from tumor removal, tissue-processing and paraffin-embedding methods, and sample storage as well as RNA extraction methods [43]. Nevertheless, RNA has been successfully extracted from stored FFPE specimens and used for quantitative measurement of mRNA levels by quantitative reverse transcription polymerase chain reaction (qRT-PCR), microarray analysis, and next-generation sequencing with successful results [43]. In our study, we used standardized protocols for pre-analytical workflow, extraction of RNA from FFPE blocks, and RNA gene expression analysis. The majority of FFPE samples showed a RIN (RNA Integrity Number) between 2 and 4. Quantification cycle (Cq) values of the qRT-PCR amplifications were between 24 and 30 for *GAPDH* mRNA (internal control). There was no significant correlation between RIN values and Cq values, which was consistent with the results from a previous study of RIN values and its effect on qRT-PCR in FFPE samples [43].

## 4. Materials and Methods

### 4.1. Patient and Clinical Samples

This study was approved by the Institutional Review Board of Seoul St. Mary’s Hospital of the Catholic University of Korea (KC13SISI0198, valid from 1 March 2013 to 31 March 2016, for study cohort 1; KC16SISI0709, valid from 1 November 2016 to 31 October 2019, for study cohort 2). We used a series of 282 surgical resection specimens, obtained at Seoul St. Mary’s Hospital from 2009 to 2013, to investigate the prevalence and clinicopathologic significance of G870A polymorphism and *CCND1* expression at the gene and protein levels in various types of thyroid tumors. We also included a separate cohort of 58 patients with NIFTP (*n* = 34) and invasive encapsulated follicular variant of PTC (*n* = 24), diagnosed from 2014 to 2017, to validate the significance of the expression of *CCND1* mRNA and cyclin D1 protein. All histologic slides were reviewed by an endocrine pathologist. Pathologic cancer stages were categorized according to the 7th and 8th editions of the American Joint Committee on Cancer (AJCC) staging manual. Recurrence risk was evaluated using the American Thyroid Association classification for risk of recurrence [44].

### 4.2. Isolation of Nucleic Acids

Genomic DNA and total RNA were isolated from formalin-fixed paraffin-embedded specimens using a RecoverAll Total Nucleic Acid Isolation kit (Ambion, Austin, TX, USA) according to the manufacturer’s protocol. Briefly, microdissected samples from deparaffinized slides were digested with a protease for 60 min at 50 °C, then for 15 min at 80 °C for total RNA isolation, and for 16 to 48 h at 50 °C for isolation of genomic DNA. Nucleic acid was isolated by capture on a glass-fiber filter. The DNase mix and RNase mix included in the kit were used for the isolation of RNA and DNA, respectively. RNA was eluted using elution solution at room temperature and DNA was eluted using nuclease-free water preheated to 95 °C.

### 4.3. Genotyping of CCND1 G870A Polymorphism

Genomic DNA was analyzed with polymerase chain reaction (PCR) analysis using specific primers, as shown in Supplementary Table S2. Polymorphism was evaluated using endpoint real-time PCR after validation by Sanger sequencing. We used a FastStart Essential DNA Probes Master containing FastStart Taq DNA Polymerase for hot start PCR (Roche Applied Science, Barcelona, Spain). The procedure was conducted using a LightCycler 96 real-time PCR system (Roche Applied Science). The probes for the alleles A and G were labeled with fluorescent HEX dye and fluorescent FAM dye, respectively, to identify the *CCND1* polymorphism at end point real-time PCR. Genotype was evaluated on a LightCycler^®^ 96 Software version 1.1.0.1320 (Roche Diagnostics GmbH, Mannheim, Germany).

### 4.4. Quantitative Real-Time PCR for CCND1 Alternative Transcripts

Total RNA was reverse-transcribed using First Strand cDNA Synthesis kit (Roche Applied Science). Quantitative real-time PCR was performed on a LightCycler 96 Real-Time PCR System (Roche Applied Science) using FastStart Essential DNA Probes Master (Roche Applied Science). Gene-specific primers and probes were designed to recognize transcripts *CCND1a* and *CCND1b*, as shown in Supplementary Table S2. The probes were selected from the Universal Probe Library (Roche Applied Science). Quantitative real-time PCR for glyceraldehyde-3-phosphate dehydrogenase (GAPDH) as reference gene to normalize the mRNA levels of *CCND1* was performed simultaneously using the same conditions.

### 4.5. Molecular Analysis of BRAF, NRAS, HRAS, and KRAS Genes

Exon 15 of the BRAF gene, exon 3 of the NRAS and HRAS genes, and exons 2 and 3 of the KRAS gene were amplified via PCR using specific primers, as shown in Supplementary Table S3. Sanger sequencing of PCR amplicons was performed using PCR primers described as previously described [37].

### 4.6. Antibody Preparation

The rabbit monoclonal cyclin D1 (clone SP4, Cat. No. 241R-18) antibody for in vitro diagnostic use was purchased from Cell Marque (Rocklin, CA, USA). A rabbit polyclonal antibody to cyclin D1b was generated using intron 4 cyclin D1b specific C-terminal peptides from Abfrontier (Seoul, Korea). Antiserum was collected after primary immunization (1 mg antigen/rabbit in Complete Freund’s Adjuvant) and three boosters (500 µg antigen/rabbit in Incomplete Freund’s Adjuvant). Rabbit immunization protocol is shown in Supplementary Table S4. The polyclonal antibody against cyclin D1b was purified from the rabbit immune serum using Protein A purification. We used Western blotting to assess antibody specificity by the presence of a single band at the expected molecular weight of 31 kDa in thyroid cancer cell lines, as shown in Supplementary Figure S2.

### 4.7. Western Blotting for Assessing Antibody Specificity

Human thyroid cancer cell lines were homogenized in radio-immunoprecipitation assay buffer (Thermo Scientific, Fremont, CA, USA) and a mixture of 5 mM EDTA and protease inhibitors (Thermo Scientific). Protein extracts were separated on 10% SDS-PAGE gels and transferred to polyvinylidene difluoride membrane (Merck Millipore, Darmstadt, Germany). After blocked in Starting Block T20 (TBS) Blocking buffer (Thermo Scientific), the membrane was incubated with primary antibodies (cyclin D1a, 1:1500; cyclin D1b, 1:1000) for 16 h at 4 °C on an orbital shaker. Primary antibodies were diluted in the blocking buffer. The membrane was washed three times for 10 min with Tris-buffered saline with Tween 20 (TBST, Thermo Scientific), followed by incubation with goat anti-rabbit IgG horseradish peroxidase conjugated antibody (1:2500, Santa Cruz Biotechnology, CA, USA) for 60 min at room temperature (22–25 °C) on an orbital shaker. The detection of immunoreaction was achieved using an enhanced SuperSignal West Pico Chemiluminescent Substrate kit (Thermo Scientific). Immuno-reactive bands were visualized using the PXi Touch Imaging System (Syngene, Frederick, MD, USA) and quantified using the GeneTools Analysis Software (Syngene).

### 4.8. Immunohistochemistry

Formalin-fixed paraffin-embedded 4-μm-thick whole-tumor sections were deparaffinized and rehydrated. Endogenous peroxidase activity was blocked by incubating the tissue sections in 3% hydrogen peroxide for 15 min at room temperature. Cyclin D1a immunostaining was performed using an automated Ventana Benchmark system (Ventana Medical Systems, Tucson, AZ, USA) in accordance with the manufacturer’s instructions. Antigen retrieval was performed for 48 min using the Ventana Benchmark CC1 standard program. Tissue sections were incubated with primary rabbit anti-cyclin D1a monoclonal antibody (pre-diluted by supplier, clone SP4, Cell Marque) for 16 min at 37 °C, followed by visualization with Ventana OptiView DAB IHC Detection Kit (OptiView HQ Linker for 8 min and OptiView HRP Multimer for 8 min) and OptiView Amplification Kit. The specimens were then counterstained with Ventana Hematoxylin II for 4 min, followed by bluing reagent (Ventana) for 4 min. Cyclin D1b immunostaining was manually performed using a Polink-2 plus HRP Rabbit DAB kit (GBI Labs, Mukilteo, WA, USA) according to the manufacturer’s instructions. Antigen retrieval for cyclin D1b was conducted by heating the slides containing the sections in 0.01 mol/L citrate buffer (pH 6.0) for 20 min using an electric pressure cooker. Tissue sections were incubated with primary rabbit anti-cyclin D1b polyclonal antibody (5 µg/mL, Abfrontier) for 30 min at room temperature. Slides were incubated with HRP Polymer-anti-Rabbit IgG (GBI Labs) for 15 min at room temperature in a humidified chamber. The antibody-antigen interaction was visualized using 3,3′-diaminobenzidine tetrahydrochloride. Sections were then counterstained with Harris’s hematoxylin (YD diagnostics, Seoul, Korea). The negative control used nonspecific rabbit IgG in place of the primary antibody. The immunostained slides were assessed in a blinded manner by a thyroid pathologist. Cyclin D1a positivity was defined as moderate to strong nuclear staining in 10% or more of tumor cells. For cyclin D1b, nuclear and cytoplasmic immunostaining was separately evaluated and considered positive if ≥10% of tumor cells showed moderate to strong expression of cyclin D1b [25].

### 4.9. The Cancer Genome Atlas Data Analysis for CCND1

We mined the Cancer Genome Atlas (TCGA) database for data on mutations and normalized mRNA expression of *CCND1* in PTC; data were downloaded from the cBioPortal website (http://www.cbioportal.org). Clinicopathologic data were downloaded from the Genomic Data Commons Data Portal website (https://portal.gdc.cancer.gov).

### 4.10. Statistical Analysis

Association between expression levels of *CCND1* mRNA isoforms and rs9344 genotypes or types of thyroid tumors was analyzed using Student’s *t*-test (normally distributed variables) or Mann–Whitney *U*-test (non-normally distributed variables) for comparing two unpaired groups. A Kolmogorov–Smirnov’s test for normal distribution was used to decide whether to apply the parametric or nonparametric test. Values of mRNA expression were presented as median and interquartile range. Correlation between clinicopathologic features and relative expression of *CCND1* mRNA isoforms and cyclin D1b protein were analyzed by the chi-square test (parametric) or the Fisher’s exact (non-parametric) where appropriate. All statistical values were calculated using Prism (version 6.05, GraphPad Software, La Jolla, CA, USA) and statistical software program SPSS (version 21.0, IBM Corp, Armonk, NY, USA). P values of less than 0.05 were considered to indicate statistically significant differences.

## 5. Conclusions

The AA genotype of the rs9344 polymorphism was associated with the overexpression of cyclin D1b in thyroid cancer. The expression of cyclin D1b (the oncogenic form) can be an effective surrogate marker for differentiating among the types of thyroid tumors and for predicting prognosis for patients with PTC. Immunostaining for cyclin D1b can improve the diagnostic accuracy of morphologically-based interpretation of NIFTP. Positive immunostaining for cyclin D1a (wild-type form) favors a neoplastic over a hyperplastic nodule, but has no diagnostic or prognostic value in NIFTP and PTC.

## Figures and Tables

**Table 1:** Expression of cyclin D1a and cyclin D1b in thyroid tumors from cohort 1 and cohort 2.

Tumor Type	*n*	Cyclin D1a	Cyclin D1b	
		Nuclear	Nuclear	Cytoplasmic
**Cohort 1**	281			
Nodular hyperplasia	16	0	0	0
Follicular adenoma	33	8 (33%)	0	0
NIFTP	9	9 (100%)	1 (11%)	0
Papillary thyroid carcinoma	170	170 (100%)	110 (64.7%)	124 (72.9%)
Classic	125	125 (100%)	83 (66.4%)	93 (74.4%)
Follicular variant	12	12 (100%)	3 (25%)	3 (25%)
Tall cell variant	33	33 (100%)	24 (73%)	28 (85%)
Follicular thyroid carcinoma	32	32 (100%)	14 (13%)	15 (16%)
Poorly differentiated thyroid carcinoma	4	4 (100%)	2 (50%)	4 (100%)
Medullary thyroid carcinoma	17	17 (100%)	12 (71%)	12 (71%)
**Cohort 2**	58			
NIFTP	34	34 (100%)	5 (15%)	4 (12%)
Invasive encapsulated follicular variant of papillary thyroid carcinoma	24	24 (100%)	9 (38%)	7 (29%)

**Table 2:** Correlation between clinicopathologic features and expression of *CCND1* mRNA isoforms and cyclin D1b protein in 170 patients with papillary thyroid carcinoma. AJCC: American Joint Committee on Cancer.

Characteristic	*n*	High Expression of *CCND1* mRNA Isoforms				High Expression of Cyclin D1b Protein			
		*CCND1a*	*p*-Value	*CCND1b*	*p*-Value	Nuclear	*p*-Value	Cytoplasmic	*p*-Value
Age (years)			0.042		0.660		0.422		0.801
<55	127	57 (44.9%)		63 (49.6%)		80 (63.0%)		92 (72.4%)	
≥55	43	27 (62.8%)		23 (53.5%)		30 (69.8%)		32 (74.4%)	
Gender			0.396		0.916		0.423		0.208
Female	133	68 (51.1%)		67 (50.4%)		84 (63.2%)		94 (70.7%)	
Male	37	16 (43.2%)		19 (51.4%)		26 (70.3%)		30 (81.1%)	
Primary tumor size (cm)			0.070		0.268		0.357		0.149
≤1.0	93	40 (42.1%)		43 (45.3%)		60 (63.2%)		66 (69.5%)	
1.0–2.0	49	27 (55.1%)		29 (59.2%)		30 (61.2%)		35 (71.4%)	
>2.0	26	17 (65.4%)		14 (53.8%)		20 (76.9%)		23 (88.5%)	
Histologic variant			0.000		0.668		0.009		0.000
Classic	125	47 (37.6%)		61 (48.8%)		83 (66.4%)		93 (74.4%)	
Follicular variant	12	9 (75.0%)		6 (50.0%)		3 (25.0%)		3 (25.0%)	
Tall cell variant	33	28 (84.8%)		19 (57.6%)		24 (72.7%)		28 (84.8%)	
Extrathyroidal extension			0.219		0.633		0.516		0.150
Absent	75	32 (42.7%)		35 (46.7%)		47 (62.7%)		51 (68.0%)	
Microscopic	81	43 (53.1%)		44 (54.3%)		52 (64.2%)		60 (74.1%)	
Gross	14	9 (64.3%)		7 (50.0%)		11 (78.6%)		13 (92.9%)	
Lymph node metastasis			0.638		0.047		0.002		0.000
Absent	80	38 (47.5%)		34 (42.5%)		42 (52.5%)		48 (60.0%)	
Present	90	46 (51.1%)		52 (57.8%)		68 (75.6%)		76 (84.4%)	
Lateral lymph node metastasis			0.152		0.613		0.059		0.010
Absent	137	64 (46.7%)		68 (49.6%)		84 (61.3%)		94 (68.6%)	
Present	33	20 (60.6%)		18 (54.5%)		26 (78.8%)		30 (90.9%)	
Distant metastasis			0.028		0.059		0.163		0.325
Absent	165	79 (47.9%)		81 (49.1%)		105 (63.6%)		119 (72.1%)	
Present	5	5 (100%)		5 (100%)		5 (100%)		5 (100%)	
*BRAF* V600E mutation			0.276		0.893		0.108		0.323
Negative	29	17 (58.6%)		15 (51.7%)		15 (51.7%)		19 (65.5%)	
Positive	141	67 (47.5%)		71 (50.4%)		95 (67.4%)		105 (74.5%)	
Recurrence risk			0.060		0.286		0.043		0.010
Low	55	20 (36.4%)		23 (41.8%)		32 (58.2%)		35 (63.6%)	
Intermediate	84	46 (54.8%)		46 (54.8%)		52 (61.9%)		60 (71.4%)	
High	31	18 (58.1%)		17 (54.8%)		26 (83.9%)		29 (93.5%)	
AJCC stage, 7th edition			0.233		0.001		0.047		0.303
I	97	44 (45.4%)		39 (40.2%)		57 (58.8%)		66 (68.0%)	
II	3	2 (66.7%)		2 (66.7%)		2 (66.7%)		2 (66.7%)	
III	67	35 (52.2%)		42 (62.7%)		48 (71.6%)		53 (79.1%)	
IV	3	3 (100%)		3 (100%)		3 (100%)		3 (100%)	
AJCC stage, 8th edition			0.022		0.011		0.081		0.282
I	149	69 (46.3%)		70 (47.0%)		93 (62.4%)		107 (71.8%)	
II	19	13 (68.4%)		14 (73.7%)		15 (78.9%)		15 (78.9%)	
IV	2	2 (100%)		2 (100%)		2 (100%)		2 (100%)	

**Table 3:** Correlation between clinicopathologic features and expression of mRNA *CCND1* isoforms and cyclin D1b protein in 125 patients with classic variant of papillary thyroid carcinoma.

Characteristic	*n*	High Expression of *CCND1* mRNA Isoforms				High Expression of Cyclin D1b Protein			
		*CCND1a*	*p*-Value	*CCND1b*	*p*-Value	Nuclear	*p*-Value	Cytoplasmic	*p*-Value
Age (years)			0.022		0.438		0.289		0.358
<55	94	30 (31.9%)		44 (46.8%)		60 (63.8%)		68 (72.3%)	
≥55	31	17 (54.8%)		17 (54.8%)		23 (74.2%)		25 (80.6%)	
Gender			0.499		0.885		0.122		0.040
Female	97	38 (39.2%)		47 (48.5%)		61 (62.9%)		68 (70.1%)	
Male	28	9 (32.1%)		14 (50.0%)		22 (78.6%)		25 (89.3%)	
Primary tumor size (cm)			0.709		0.078		0.473		0.156
≤1.0	80	28 (35.0%)		33 (41.3%)		51 (63.8%)		56 (70.0%)	
1.0–2.0	30	13 (43.3%)		19 (63.3%)		20 (66.7%)		23 (76.7%)	
>2.0	15	6 (40.0%)		9 (60.0%)		12 (80.0%)		14 (93.3%)	
Extrathyroidal extension			0.206		0.084		0.823		0.411
Absent	59	19 (32.2%)		24 (40.7%)		38 (64.4%)		41 (69.5%)	
Microscopic	58	24 (41.4%)		32 (55.2%)		39 (67.2%)		45 (77.6%)	
Gross	8	4 (50.0%)		5 (62.5%)		6 (75.0%)		7 (87.5%)	
Lymph node metastasis			0.661		0.038		0.006		0.005
Absent	59	21 (35.6%)		23 (39.0%)		32 (54.2%)		37 (62.7%)	
Present	66	26 (39.4%)		38 (57.6%)		51 (77.3%)		56 (84.8%)	
Lateral lymph node metastasis			0.142		0.144		0.202		0.065
Absent	99	34 (34.3%)		45 (45.5%)		63 (63.6%)		70 (70.7%)	
Present	26	13 (50.0%)		16 (61.5%)		20 (76.9%)		23 (88.5%)	
Distant metastasis			0.007		0.059		0.167		0.327
Absent	120	42 (35.0%)		81 (49.1%)		78 (65.0%)		88 (73.3%)	
Present	5	5 (100%)		5 (100%)		5 (100%)		5 (100%)	
*BRAF* V600E mutation			0.743		0.713		0.694		0.234
Negative	17	7 (41.2%)		9 (52.9%)		12 (70.6%)		15 (88.2%)	
Positive	108	40 (37.0%)		52 (48.1%)		71 (65.7%)		78 (72.2%)	
Recurrence risk			0.258		0.056		0.190		0.111
Low	48	14 (29.2%)		17 (35.4%)		30 (62.5%)		33 (68.8%)	
Intermediate	54	22 (40.7%)		30 (55.6%)		34 (63.0%)		39 (72.2%)	
High	23	11 (47.8%)		14 (60.9%)		19 (82.6%)		21 (91.3%)	
AJCC stage, 7th edition			0.014		0.007		0.065		0.025
I	70	20 (28.6%)		27 (38.6%)		42 (60.0%)		47 (67.1%)	
II	3	2 (66.7%)		2 (66.7%)		2 (66.7%)		2 (66.7%)	
III	47	22 (44.9%)		29 (59.2%)		36 (73.5%)		41 (83.7%)	
IV	3	3 (100%)		3 (100%)		3 (100%)		3 (100%)	
AJCC stage, 8th edition			0.004		0.008		0.038		0.131
I	107	35 (32.7%)		47 (43.9%)		67 (62.6%)		77 (72.0%)	
II	16	10 (62.5%)		12 (75.0%)		14 (87.5%)		14 (87.5%)	
IV	2	2 (100%)		2 (100%)		2 (100%)		2 (100%)	

**Table 4:** Expression of *CCND1b* mRNA and cyclin D1b protein in an independent cohort of noninvasive follicular thyroid neoplasm with papillary-like nuclear features (NIFTP) and invasive encapsulated follicular variant of papillary thyroid carcinoma.

Molecular Alteration	NIFTP (*n* = 34)	Invasive Encapsulated Follicular Variant of Papillary Thyroid Carcinoma (*n* = 24)	*p*-Value
High expression of *CCND1b* mRNA	18 (52.9%)	13 (54.2%)	0.927
High expression of nuclear cyclin D1b	5 (14.7%)	9 (37.5%)	0.046
High expression of cytoplasmic cyclin D1b	4 (11.8%)	7 (29.2%)	0.096
*BRAF* V600E mutation	0	0	
*BRAF* K601E mutation	2 (5.9%)	0	0.339
*RAS* mutation (total)	20 (58.8%)	13 (54.2%)	0.724
*NRAS* mutation	14 (41.2%)	8 (33.3%)	0.544
*HRAS* mutation	6 (17.6%)	4 (16.7%)	0.605
*KRAS* mutation	0	2 (8.3%)	0.167

## Supplementary Materials

The following are available online at http://www.mdpi.com/2072-6694/10/11/437/s1, Figure S1: Hürthle cells in Hashimoto’s thyroiditis are positive for cyclin D1a (a, × 400) and negative for cyclin D1b (b, ×400). Most chronic inflammatory cells mixed with Hürthle cells are negative for cyclin D1a and positive for cyclin D1b. Parathyroid cells are positive for cyclin D1a (c, ×400) and negative for cyclin D1b (d, ×400). Figure S2: The rabbit polyclonal cyclin D1b antibody was derived from the intron 4 sequence (VSEGDVPGSLAGAYRGRHLVPRKCRGWCQGPQG). This rabbit polyclonal cyclin D1b antibody was used in Western blot to detect cyclin D1b in two thyroid cancer cell lines, SNU80 (anaplastic thyroid carcinoma) with *BRAF* G469R mutation and SNU790 (papillary thyroid carcinoma) with *BRAF* V600E mutation, which were obtained from the Korea Cell Line Bank (Seoul National University, Seoul, Korea). Cyclin D1b runs as a 31 kDa molecule on 10% SDS-PAGE gel. Table S1: Correlation between clinicopathologic features and expression of *CCND1* mRNA in The Cancer Genome Atlas (TCGA) dataset of papillary thyroid carcinoma. Table S2: Sequences of primers and probes for the analysis of *CCND1* gene. Table S3: Sequences of primers for the molecular analysis of *BRAF*, *NRAS*, *HRAS*, and *KRAS* genes. Table S4: Rabbit immunization protocol for polyclonal cyclin D1b antibody production.
