# Supplementary material for: CCND1 Splice Variant as A Novel Diagnostic and Predictive Biomarker for Thyroid Cancer
Source: Cancers (Basel). 2018 Nov 13;10(11):437. doi: 10.3390/cancers10110437 (PMC6266131; doi:10.3390/cancers10110437)
Supplement: Supplementary file 1 [file cancers-10-00437-s001.pdf]

Supplementary Materials: CCND1 Splice Variant as a Novel Diagnostic and Predictive Biomarker for Thyroid Cancer

Sora Jeon, Yourha Kim, Young Mun Jeong, Ja Seong Bae and Chan Kwon Jung

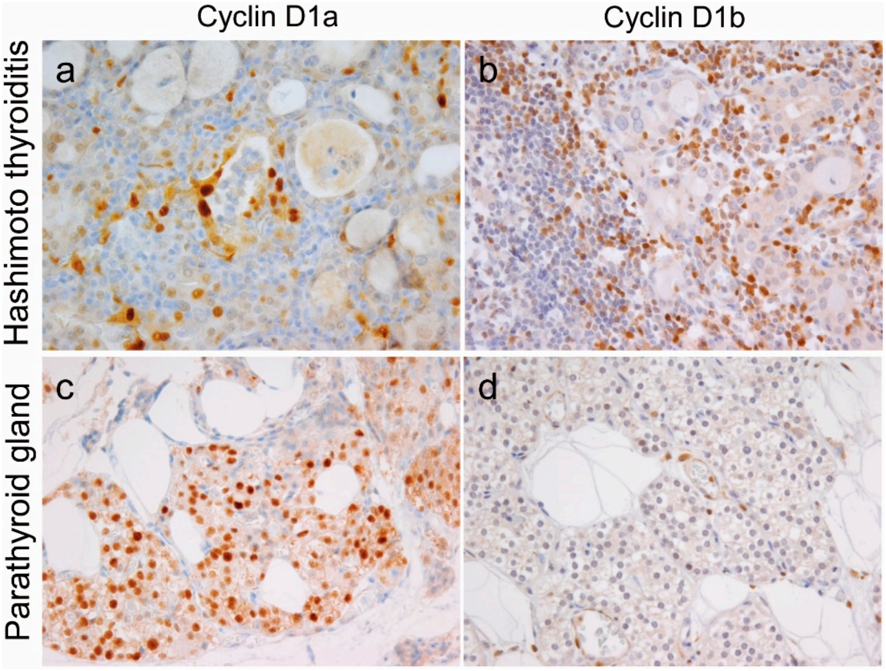

Figure S1. Hürthle cells in Hashimoto's thyroiditis are positive for cyclin D1a (**a**, × 400) and negative for cyclin D1b (**b**, ×400). Most chronic inflammatory cells mixed with Hürthle cells are negative for cyclin D1a and positive for cyclin D1b. Parathyroid cells are positive for cyclin D1a (**c**, ×400) and negative for cyclin D1b (**d**, ×400).

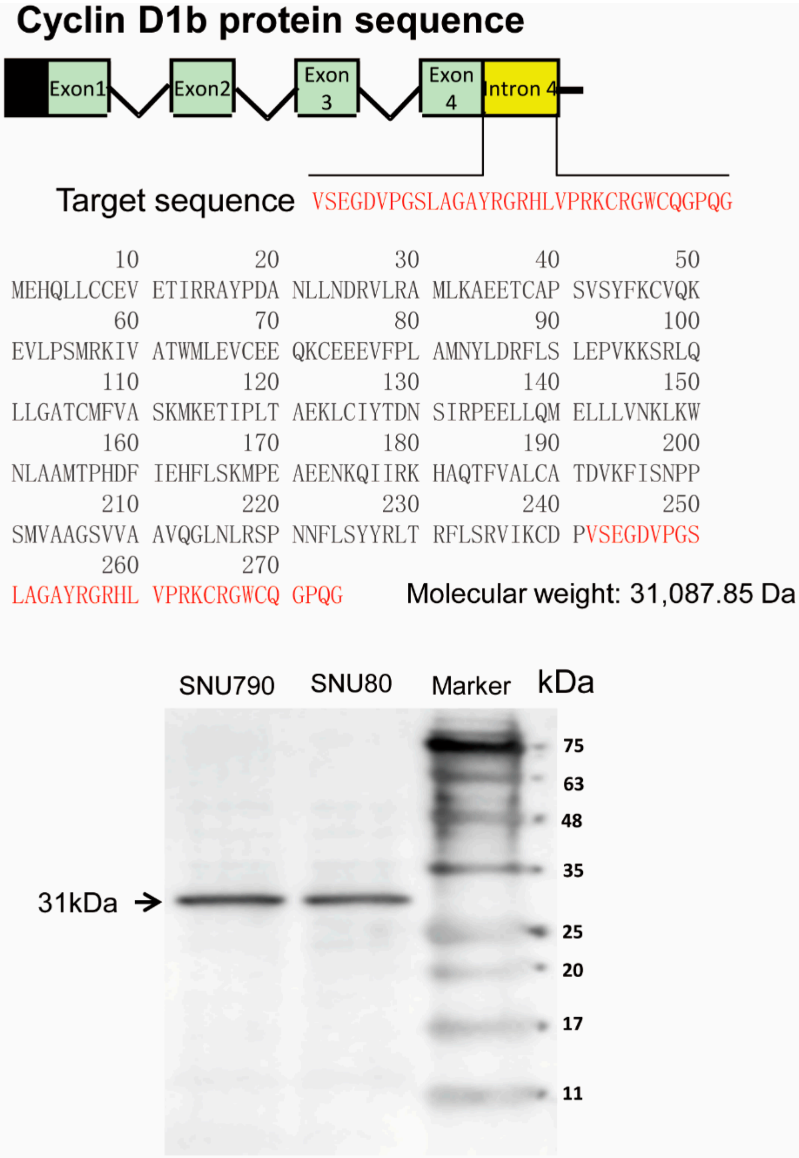

Figure S2. The rabbit polyclonal cyclin D1b antibody was derived from the intron 4 sequence (VSEGDVPGSLAGAYRGRHLVPRKCRGWCQGPQG). This rabbit polyclonal cyclin D1b antibody was used in Western blot to detect cyclin D1b in two thyroid cancer cell lines, SNU80 (anaplastic thyroid carcinoma) with *BRAF* G469R mutation and SNU790 (papillary thyroid carcinoma) with *BRAF* V600E mutation, which were obtained from the Korea Cell Line Bank (Seoul National University, Seoul, Korea). Cyclin D1b runs as a 31kDa molecule on 10% SDS-PAGE gel.

Table S1. Correlation between clinicopathologic features and expression of *CCND1* mRNA in The Cancer Genome Atlas (TCGA) dataset of papillary thyroid carcinoma.

Characteristic	Low Expression of *CCND1* mRNA	High Expression of *CCND1* mRNA	*p*-value
Age (years)			
<45	101 (47.9%)	110 (52.1%)	0.397
≥45	126 (51.9%)	117 (48.1%)	
Gender			
Male	159 (48.0%)	172 (52.0%)	0.17
Female	68 (55.3%)	55 (44.7%)	
Histologic type			
Classic	148 (47.4%)	164 (52.6%)	0.360
Follicular	59 (59.6%)	40 (40.4%)	
Tall cell	15 (44.1%)	19 (55.9%)	
Other	5 (55.6%)	4 (44.4%)	
Extrathyroidal extension			
None	161 (52.1%)	148 (47.9%)	0.284
Minimal (T3)	51 (43.6%)	66 (56.4%)	
Moderate/advanced (T4)	7 (46.7%)	8 (53.3%)	
Pathologic T (pT) stage			
pT1	77 (58.8%)	54 (41.2%)	0.09
pT 2	67 (44.1%)	85 (55.9%)	
pT 3	72 (47.7%)	79 (52.3%)	
pT 4	9 (50.0%)	9 (50.0%)	
Pathologic lymph node (pN) stage			
pN0	105 (50.5%)	103 (49.5%)	0.664
pN1	97 (48.3%)	104 (51.7%)	
pNX	25 (55.6%)	20 (44.4%)	
Metastasis (M) stage			
M0	116 (47.9%)	126 (52.1%)	0.371
M1	4 (50.0%)	4 (50.0%)	
MX	106 (52.2%)	97 (47.8%)	
American Joint Committee on Cancer tumor stage			
Stage I	131 (50.4%)	129 (49.6%)	0.974
Stage II	25 (51.0%)	24 (49.0%)	
Stage III	47 (48.0%)	51 (52.0%)	
Stage IV	23 (51.1%)	22 (48.9%)	
BRAF-RAS molecular type			
BRAF-like	111 (40.8%)	161 (59.2%)	<0.001
RAS-like	81 (68.1%)	38 (31.9%)	
Recurrence risk group			
Low	90 (54.2%)	76 (45.8%)	0.269
Intermediate	117 (46.4%)	135 (53.6%)	
High	13 (54.2%)	11 (45.8%)	

The mRNA expression levels of the *CCND1*mRNA were grouped as high or low based on the median value.

Table S2. Sequences of primers and probes for the analysis of CCND1 gene.

Name	Position	Sequence (5′→3′)
**Endpoint real-time PCR for detection of CCND1 G/A870 polymorphism**		
CCND1 Forward	Exon 4	CTTCCTGTCCTACTACCG
CCND1 Reverse	Intron 4	GTGTCTCCCCCTGTAAG
CCND1 Probe_A	Exon 4-Intron4	[HEX]CCTCACTTACTGGGTCACACT[BHQ1]
CCND1 Probe_G	Exon 4-Intron4	[6FAM]CCTCACTTACCGGGTCACACT[BHQ1]
**Sanger sequencing for CCND1 G/A870 polymorphism**		
CCND1 Forward	Exon 4	AGTTCATTTCCAATCCGCCC
CCND1 Reverse	Intron 4	TTTCCGTGGCACTAGGTGTC
**Detecting mRNA expression of CCND1 isoforms by quantitative real-time PCR**		
CCND1a Forward	Exon 4	GTCCTACTACCGCCTCACACG
CCND1a Reverse	Exon 5	TTCGATCTGCTCCTGGCAG
CCND1b Forward	Exon 4	TGAGGAGCCCCAACAACTTC
CCND1b Reverse	Intron 4	CCTGGGACATCACCCTCACTTA
CCND1a/1b Probe	Exon 4	[6FAM]TTCCTCTCCAGAGTGATCAAGTGTGACCC[BHQ1]
GAPDH Forward	Exon 3	AGTGGATATTGTTGCCATC
GAPDH Reverse	Exon 4	TTCCATTGATGACAAGCTT
GAPDH Probe	Exon 4	[6FAM]ATGGGTGGAATCATATTGGAACAT[BHQ1]

Table S3. Sequences of primers for the molecular analysis of *BRAF*, *NRAS*, *HRAS*, and *KRAS* genes.

Name	Forward	Reverse
BRAF	5′-TCATAATGCTTGCTCTGATAGGA-3′	5′-GGCCAAAAATTTAATCAGTGGA-3′
NRAS(codon61)	5′-CCCCTTACCCTCCACACC-3′	5′-GAGGTTAATATCCGCAAATGACTT-3′
NRAS(codon61)	5′-GTGAAACCTGTTTGTTGGAC-3′	5′-CCTGTAGAGGTTAATATCCG-3′
NRAS(codon61)	5′-ACACCCCCAGGATTCTTACAG-3′	5′-GCCTGTCCTCATGTATTGGTC-3′
HRAS(exon3)	5′-GTCCTCCTGCAGGATTCCTA-3′	5′-CGGGGTTCACCTGTACT-3′
KRAS(exon2)	5′-GGTGAGTTTGTATTAAAAGGTACTGG-3′	5′TCCTGCACCAGTAATATGCA-3′
KRAS(exon3)	5′-GGTGCACTGTAATAATCCAGAC-3′	5′TGATTTAGTATTATTTATGGC-3′

Table S4. Rabbit immunization protocol for polyclonal cyclin D1b antibody production.

Procedure	Protocol week	Description	Note
Pre-immune serum collection	Week 0	Bleed; Control serum	~ 1 mL serum/rabbit
Primary injection of antigen	Week 0	Immunize with 1mg antigen/rabbit in Complete Freund's Adjuvant	Subcutaneous injection
First booster	Week 4	Immunize with 500 μg/rabbit antigen in IFA	Subcutaneous injection
First serum collection	Week 5	Bleed; peptide-specific ELISA titration of antibody	~ 1 mL serum/rabbit
Second booster	Week 6	Immunize with 500 μg/rabbit antigen in IFA	Subcutaneous injection
Second serum collection	Week 7	Bleed; peptide-specific ELISA titration of antibody	~ 1 mL serum/rabbit
Third booster	Week 8	Immunize with 500 μg/rabbit antigen in IFA	Subcutaneous injection
Third serum collection	Week 9	Sacrifice (Heart puncture); peptide-specific ELISA titration of antibody	~ 50 mL serum/rabbit

IFA, Incomplete Freund's Adjuvant.

© 2018 by the authors. Licensee MDPI, Basel, Switzerland. This article is an open access article distributed under the terms and conditions of the Creative Commons Attribution (CC BY) license (http://creativecommons.org/licenses/by/4.0/).
